# *gldc* Is Essential for Renal Progenitor Patterning during Kidney Development

**DOI:** 10.3390/biomedicines10123220

**Published:** 2022-12-12

**Authors:** Nicole E. Weaver, Allison Healy, Rebecca A. Wingert

**Affiliations:** Department of Biological Sciences, Center for Stem Cells and Regenerative Medicine, Center for Zebrafish Research, Boler-Parseghian Center for Rare and Neglected Diseases, Warren Center for Drug Discovery, University of Notre Dame, Notre Dame, IN 46556, USA

**Keywords:** kidney, nephron, nonketotic hyperglycinemia, NKH, *gldc*, glycine

## Abstract

The glycine cleavage system (GCS) is a complex located on the mitochondrial membrane that is responsible for regulating glycine levels and contributing one-carbon units to folate metabolism. Congenital mutations in GCS components, such as *glycine decarboxylase* (*gldc*), cause an elevation in glycine levels and the rare disease, nonketotic hyperglycinemia (NKH). NKH patients suffer from pleiotropic symptoms including seizures, lethargy, mental retardation, and early death. Therefore, it is imperative to fully elucidate the pathological effects of *gldc* dysfunction and glycine accumulation during development. Here, we describe a zebrafish model of *gldc* deficiency that recapitulates phenotypes seen in humans and mice. *gldc* deficient embryos displayed impaired fluid homeostasis suggesting renal abnormalities, as well as aberrant craniofacial morphology and neural development defects. Whole mount in situ hybridization (WISH) revealed that *gldc* transcripts were highly expressed in the embryonic kidney, as seen in mouse and human repository data, and that formation of several nephron segments was disrupted in *gldc* deficient embryos, including proximal and distal tubule populations. These kidney defects were caused by alterations in renal progenitor populations, revealing that the proper function of Gldc is essential for the patterning of this organ. Additionally, further analysis of the urogenital tract revealed altered collecting duct and cloaca morphology in *gldc* deficient embryos. Finally, to gain insight into the molecular mechanisms underlying these disruptions, we examined the effects of exogenous glycine treatment and observed analogous renal and cloacal defects. Taken together, these studies indicate for the first time that *gldc* function serves an essential role in regulating renal progenitor development by modulating glycine levels.

## 1. Introduction

Nonketotic hyperglycinemia (NKH) is a rare disease that affects 1:76,000 people, causing one-third of patients to succumb to the disease in their first year of life [1,2]. NKH patients experience a wide range of symptoms such as hypotonia, lethargy, seizures, coma, developmental delays, and apnea that occur at varying severities [3]. The disease is caused by a defect in the glycine cleavage system (GCS), a complex of four proteins that function to break down glycine into one-carbon units to be later utilized in folate metabolism. While NKH can be caused by a defect in any of the GCS enzymes, ~75% of cases are caused by *glycine decarboxylase* (GLDC) deficiency [4]. There are treatments to alleviate symptoms of the disease, but none can resolve the developmental defects patients experience [5]. Depending on where the mutation is located on GLDC and what type of mutations occurs, patients experience a variety of symptoms of differing severities [6,7]. To better understand this variability, computational models have been used to compare disease severity across multiple genetic lesions [6,8]. Mammalian models have also been useful in analyzing *Gldc* in brain development as many patients experience neurological symptoms [9,10,11,12,13]. However, prenatal lethality in these mouse models prevents studies into the specific effects *Gldc* has on other tissues. Further, while mammals are useful models for research, their growth in utero complicates early developmental studies [14].

The zebrafish provides a reliable, efficient model to systematically delineate the role of *gldc* in early developmental processes. Zebrafish are a tractable model as they have high genetic conservation with humans and other mammals, but develop ex utero in transparent chorions. Therefore, it is possible to genetically alter zebrafish embryos and analyze them over time with a larger sample size than mice [14]. While we can study the advancement and growth of various tissues in the zebrafish, the kidney is a particularly simplistic and relevant aspect of this model [15,16]. Vertebrate kidneys are comprised of structural and functional units known as nephrons, with dozens to millions depending on the species and stage of life [17]. In the zebrafish embryo, the kidney is anatomically simple, being composed of two nephrons that share a single blood filter at the midline, followed by tubules that selectively secrete and absorb solutes running parallel down the length of the body, which reconnect at the cloaca, a shared exit way for waste [18,19,20]. The nephron tubules include various segments made of specialized epithelial cells that function to transport ions [19]. Multiple tubule segments are conserved between mammals and zebrafish [17,19,20,21,22]. Many of the genetic pathways underlying the differentiation of the cells that make these tubules remain to be elucidated. While GLDC has previously been described as important in the formation of the central nervous system (CNS), here, we demonstrate for the first time that it has an essential role in the segmental patterning of the nephron during embryonic kidney development.

## 2. Materials and Methods

### 2.1. Ethics Statement and Zebrafish Husbandry

The Freimann Life Science Center at the University of Notre Dame tended to all adult zebrafish. All WT animals used in this study were the Tübingen strain. Embryonic zebrafish were maintained in E3 medium at 28.5 °C, staged at the desired time point, anesthetized in tricaine, and fixed in 4% paraformaldehyde (PFA) [23]. The University of Notre Dame Institutional Animal Care and Use Committee (IACUC) approved and oversaw all studies under protocol numbers 19-06-5412 and 20-09-6240. Analysis of all experimental work was performed in a blinded manner.

### 2.2. Morpholino Knockdown

Morpholino oligonucleotides (MO) were designed and then obtained from GeneTools, LLC, and stored at −20 °C. The *gldc* MOs targeted two sites to block splicing: 5′–CTCTCGGAGTTGAGgtaagagctgt–3′ and 5′–ctggttatttcagTCAGTCTCTCAG–3′. Splicing was assessed using reverse transcriptase polymerase chain reaction (RT-PCR) with the following pair of primers: 5′–GAACGAATTCTGCCCAGGCACGAT–3′, 5′–GTTTGAAAATGGACGATCCAGTGT–3′. Products were isolated by PCR purification and Sanger sequencing confirmed the retention of intron 1 led to the inclusion of multiple premature stop codons. For microinjections, MOs were diluted in RNase-free water and 1–2 nanoliters were injected into the 1-cell stage.

### 2.3. capped RNA (cRNA) Synthesis and Rescue Studies

The zebrafish *gldc* ORF was cloned into a pUC57 vector. The 5′ end included an SP6 promoter, EcoRV site, SacII site, and a Kozak consensus sequence. On the 3′ end, there was a T7 promoter, Xho1 site, XbaI site, Not1 site, an SV40 PolyA tail, and a series of stop codons. To make cRNA, the vector was linearized with Xno1 and synthesized with SP6 RNA polymerase then purified and stored at −80 °C. *gldc* cRNA was co-injected with the *gldc* MOs at a concentration of 66 pg.

### 2.4. Whole-Mount In Situ Hybridization (WISH)

WISH was conducted as previously described [24,25,26]. Linearized plasmids were transcribed in vitro with T7, T3, or SP6 enzymes to create digoxigenin (DIG) or fluorescein (FLU) anti-sense RNA probes. All WISH experiments were performed in triplicate with a sample size of greater than 30 animals for each replicate. Representative samples were imaged and analyzed.

### 2.5. Dextran FITC Injections

WT and *gldc* morphants were incubated in 0.003% PTU (Sigma-Aldrich, St. Louis, MO, USA). At 48 hpf, animals were anesthetized and 40 kDa Dextran FITC conjugate (5 mg/mL) [27] was injected into the somite in order to introduce it to circulation [28]. Embryos were imaged at 6 hpi, 24 hpi, and 48 hpi. The mean fluorescent intensity of the head and pericardium was calculated in ImageJ, and the percent fluorescence was calculated utilizing the 6 hpi fluorescent intensity as a baseline.

### 2.6. Acridine Orange (AO) Assay

AO experiments were conducted as described [28,29]. AO (Sigma-Aldrich, St. Louis, MO, USA) was prepared by dissolving 50 mg in 50 mL of MilliQ water to create a 100× solution and stored at −20 °C, protected from light. AO was diluted in E3 to a 1× working solution before being applied to 24 hpf live embryos. Embryos were incubated in AO/E3 for 30 min at room temperature, then washed 3 times for 10 min with E3. Samples were anesthetized in tricaine and imaged in methylcellulose.

### 2.7. Alcian Blue Stain

Alcian Blue staining was performed as described [30]. At 4 dpf, embryos were fixed in 4% PFA overnight at 4 °C in glass vials. They were dehydrated in 100% MeOH at −20 °C, then rehydrated. Animals were bleached at room temperature for 1 h, then rinsed in PBST before being digested in 1× proteinase K (10 mg/mL) for 15 min. After another PBST rinse, animals were incubated overnight at room temperature in 0.1% Alcian Blue dissolved in 70% ethanol and 5% concentrated HCl while rocking. Zebrafish larvae were destained with acidic ethanol for 4 h on the rocker, then rinsed in PBST. The samples were dehydrated in an ethanol series and stored in glycerol before imaging.

### 2.8. Dextran-Rhodamine Brain Ventricle Injections

Brain ventricle injections were performed as described [31]. In brief, zebrafish embryos were anesthetized with 0.2% tricaine at 24 hpf. The hindbrain ventricle was injected with ~6 nL of 40 kDa dextran conjugated rhodamine (5 mg/mL) [27]. Images were taken with brightfield and fluorescence microscopy on a compound microscope and superimposed with Adobe Photoshop.

### 2.9. Glycine Treatment

Glycine (Sigma-Aldrich, St. Louis, MO, USA). After embryos reached the 50% epiboly stage, they were bathed in the appropriate glycine/E3 concentration and protected from ambient light, as described for exogenous drug treatment [32,33,34,35]. The concentration range for exposure was based on previous studies [36]. Embryos were grown up to the appropriate time point, imaged, or fixed as previously described.

### 2.10. Image Acquisition and Statistical Analysis

Live and WISH images were taken on a Nikon Eclipse Ni with a DS-Fi2 camera. Live zebrafish were imaged in methylcellulose. Fixed samples were imaged in glycerol. WISH measurements of absolute length were completed on representative samples at 10× magnification. AO^+^ and multiciliated cells were imaged at 10× and counted with the multi-point tool on ImageJ/Fiji. Cloacal area was measured at 20× on ImageJ/Fiji with the polygon selection tool. Experiments were completed in triplicate with at least 30 samples each (except brain ventricle injections, n = 3). Measurements were inputted into GraphPad Prism 9 in which averages and standard deviations (SD) were calculated. Depending on the number of treatment groups, *t*-tests or ANOVA were completed and significance was determined. The survival curve was completed in triplicate, with a sample size of 75 each.

## 3. Results

### 3.1. A Severe gldc Deficient Model Exhibits Phenotypes Consistent with Impaired Kidney Function

Previous studies reported a *gldc* deficient zebrafish model with behavioral characteristics similar to severe NKH cases [37]. However, these mutant larvae exhibited overall normal morphology [37]. This is intriguing, as data from human patients and murine *Gldc* knockout models suggest that severe physiological defects ensue across various tissues when normal expression of this gene is abrogated [9,10,11,12,13,38,39]. To expand on these previous studies, we aimed to create a severely *gldc* deficient zebrafish model recapitulating the defects observed in GLDC/*Gldc* deficient humans and mice.

**Figure 1:**
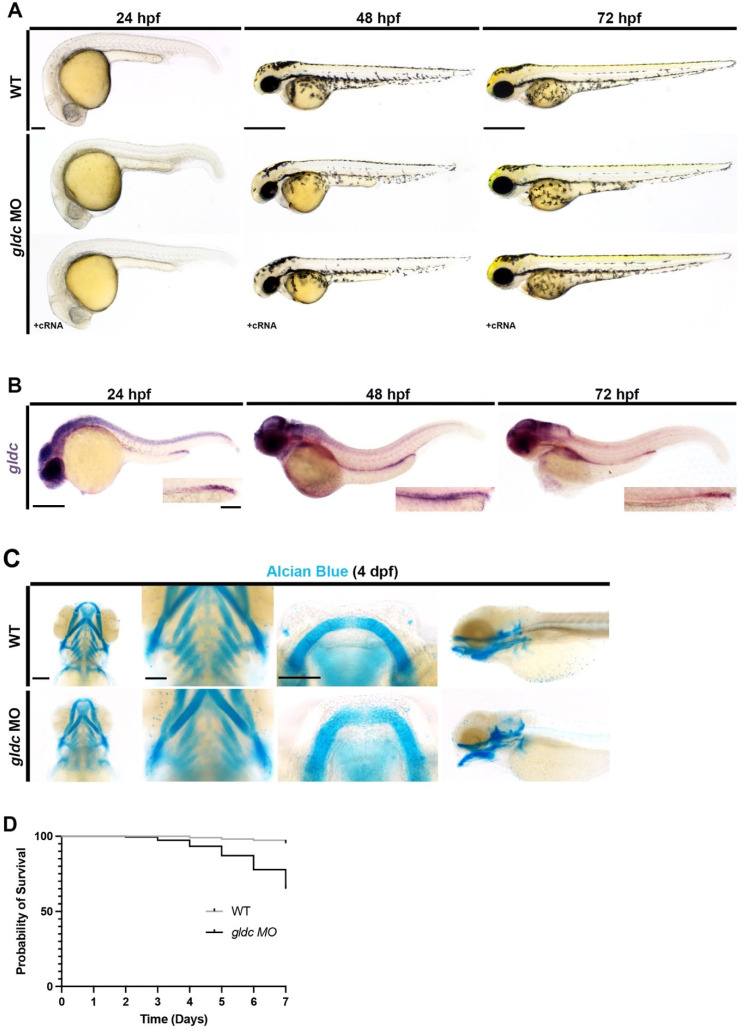
A severe *gldc* deficient model exhibits phenotypes consistent with impaired kidney function. (**A**) Live images of WT and *gldc* morphants at 24 hpf, 48 hpf, and 72 hpf. Images at 24 hpf reveal gray pallor within the head of *gldc* morphants. At 48 hpf, pericardial edema becomes evident and persists through 72 hpf. At 72 hpf, craniofacial cartilage begins to develop, and abnormalities are observed. Scale bars = 100 μM, 400 μM. (**B**) WISH of WT *gldc* expression at 24 hpf, 48 hpf, and 72 hpf reveals *gldc* transcripts within the CNS and pronephros. Scale bars = 200 μM (main image) and 50 μM (inset). (**C**) Alcian Blue cartilage staining in WT and *gldc* morphants at 4 dpf. Decreased number of pharyngeal arches and aberrant jaw morphology were seen in *gldc* morphants. Scale bars = 100 μM, 50 μM, 50 μM. (**D**) A survival curve of WT animals and *gldc* morphants reveals that *gldc* deficient animals have a decreased percent survivability over seven days.

To survey *gldc* deficiency in zebrafish, we performed a loss of function study utilizing a splice-blocking morpholino oligonucleotide (MO). Upon RT-PCR and Sanger sequencing analysis, we found the MO resulted in the inclusion of intron 1 which produced a transcript encoding multiple in-frame premature stop codons (Supplemental Figure S1A,B). Upon surveying live phenotypes, we found that *gldc* morphants exhibited gray pallor in the head at 24 h post-fertilization (hpf), which typically indicates cell death in the area (Figure 1A) [40,41]. At 48 and 72 hpf, *gldc* deficient embryos exhibited mild pericardial edema and hydrocephalus, indicating impaired kidney function (Figure 1A) [42]. These phenotypes were rescued by provision of *gldc* cRNA (Figure 1A). Compared to their wild-type (WT) siblings, *gldc* morphants had a decreased percent survivability, recapitulating the lethality noted in patients and mammalian models of NKH (Figure 1D) [9,10,11,12,13,38,39].

Previous studies have documented extensive *Gldc* expression within the CNS and the kidney of embryonic mice [43]. In humans, GLDC is heavily expressed in tubular precursor cells [44,45]. To interrogate the expression pattern in zebrafish, we utilized the whole mount in situ hybridization (WISH) on WT embryos. At 24 hpf, *gldc* transcripts localized to the CNS and distal portion of the pronephros (Figure 1B). Expression in the CNS and kidney was consistent across several time points, and at 72 hpf, *gldc* transcripts were observed within the neck of the kidney (Figure 1B). Given the conserved and consistent expression pattern over several time points, we hypothesized that *gldc* is vital for nephron segmentation patterning.

Surveying of live phenotypes at 72 hpf revealed abnormalities in the beginning stages of jaw formation in *gldc* deficient animals (Figure 1A). In some NKH patients, micrognathia (an abnormally small jaw) or retrognathia (unusual position of the mandible) were observed [46,47]. To analyze this further in our model, we utilized Alcian Blue staining at 4 dpf to visualize the craniofacial cartilage of *gldc* morphants [30]. Upon knockdown, an overall change in the structure of the jaw was observed, with obvious alterations in Meckel’s cartilage (Figure 1C). Additionally, the number of pharyngeal arches decreased in morphants (Figure 1C).

### 3.2. gldc Deficient Zebrafish Display Phenotypes Consistent with Gldc Deficient Mammals

Alterations in brain patterning, ventricle size, and neural tube development have been noted in mammalian models and humans with *gldc* deficiency [9,10,11,12]. Upon MRI imaging of the brains of NKH patients, the main phenotypes observed were ventriculomegaly and atrophy [10,11]. In *Gldc* deficient mice, some suffered from prenatal lethality due to neural tube defects, while those that survived exhibited enlarged ventricles and hydrocephalus [9,12]. We hypothesized that our morphant model would recapitulate some of the phenotypes seen in mammals.

**Figure 2:**
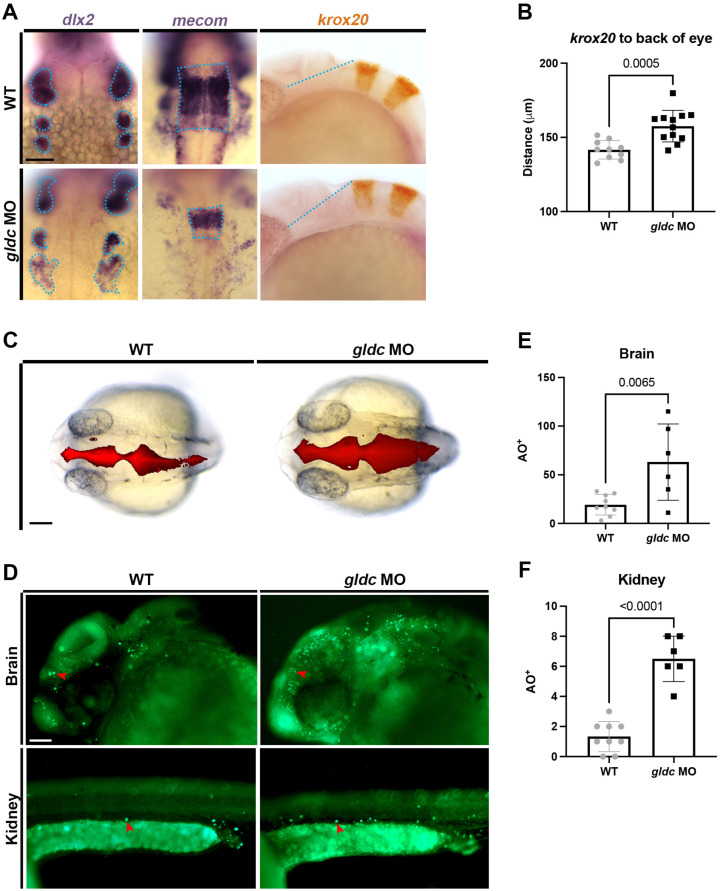
*gldc* deficient zebrafish display phenotypes consistent with *Gldc* deficient mammals. (**A**) WISH analysis of *mecom* in WT and *gldc* morphants revealed an altered rhombomere four. *gldc* morphants exhibited disorganized migrating neural crest cell populations marked with *dlx2*. *krox20*, a gene expressed in rhombomeres three and five, appeared shifted in comparison to the back of the eye. Scale bar = 50 μM. (**B**) Quantification of the distance between the edge of rhombomere three and the back of the eye. (**C**) Injections of dextran-rhodamine into the brain ventricles of live WT and *gldc* morphant animals at 24 hpf. In *gldc* morphants, brain ventricles appear enlarged. Scale bar = 50 μM. (**D**) Acridine Orange assay in WT and *gldc* morphants at 24 hpf; red arrowheads indicate example AO^+^ cells. Scale bar = 50 μM. (**E**,**F**) Quantification of AO^+^ cells in the brain and kidney, respectively. Distance measurements and cell counts were compared by unpaired T-tests. Significant differences are shown above the brackets.

First, we investigated migrating cranial neural crest cells, which migrate anteriorly to become a variety of tissues, including craniofacial cartilage and sensory ganglia [48]. The migrating neural crest cell population expressing *dlx2* appeared disorganized, which may explain the alterations observed upon Alcian Blue staining (Figure 2A). Next, we analyzed several brain markers with WISH at 24 hpf. Upon analysis of *MDS1 and EVI1 complex locus* (*mecom*), a transcription factor known to be expressed in the rhombomeres [49], we found that *gldc* morphants exhibited altered patterning (Figure 2A). To further analyze rhombomere patterning, we utilized *krox20*, which is expressed in rhombomeres three and five. We found that the distance between rhombomere three and the back of the eye was increased in *gldc* morphants (Figure 2A,B). These changes suggest that *gldc* deficiency is impacting brain development in a similar manner to reports in mice and humans [9,10,11,12].

As ventriculomegaly is one of the main phenotypes of GLDC/*Gldc* deficiency in humans and mice, we aimed to examine the ventricles in *gldc* morphants [9,10,11,12]. After anesthesia, we injected dextran-rhodamine into the brain ventricles at 24 hpf and captured images immediately after [31]. *gldc* morphants exhibited enlarged ventricles compared to their WT siblings, recapitulating mammalian phenotypes (Figure 2B). To further analyze why the changes in brain morphology were occurring, we conducted an Acridine Orange (AO) assay to mark cells undergoing apoptosis in 24 hpf animals. A count of AO-positive cells revealed increased cell death in the head region of *gldc* deficient animals (Figure 2C,D). Additionally, we noted an increase in apoptosis within the pronephros (Figure 2C,E). Overall, we concluded that our model depicted more morphologically severe cases of *gldc* deficiency, reflecting what has been observed in humans and mice. Next, we strived to dissect how the kidney was affected by *gldc* knockdown.

### 3.3. gldc Is Necessary for Proper Pronephros Segment Patterning

The zebrafish pronephros is composed of two nephrons that run parallel down the length of the body. The nephrons consist of a blood-filtering organ, the glomerulus, followed by two proximal and two distal segments [19]. Over the years, the zebrafish pronephros has provided a reliable and simplistic model to study kidney segmentation [15,16,17,20,22]. Nephron segmentation is a complex genetic process that involves the specification of transporter cells responsible for solute reabsorption and secretion [15], and occurs rapidly during zebrafish ontogeny over the first 24 hpf [19,21,49,50,51,52]. We have observed the presence of GLDC/*Gldc*/*gldc* transcripts within the developing kidney in human, mouse, and zebrafish (Figure 1C) [43,44,45]. Animals deficient in *gldc* exhibited fluid retention and increased cell death in the pronephros, suggesting kidney impairment (Figure 1A). Therefore, we hypothesized that *gldc* plays a role in pronephros development.

**Figure 3:**
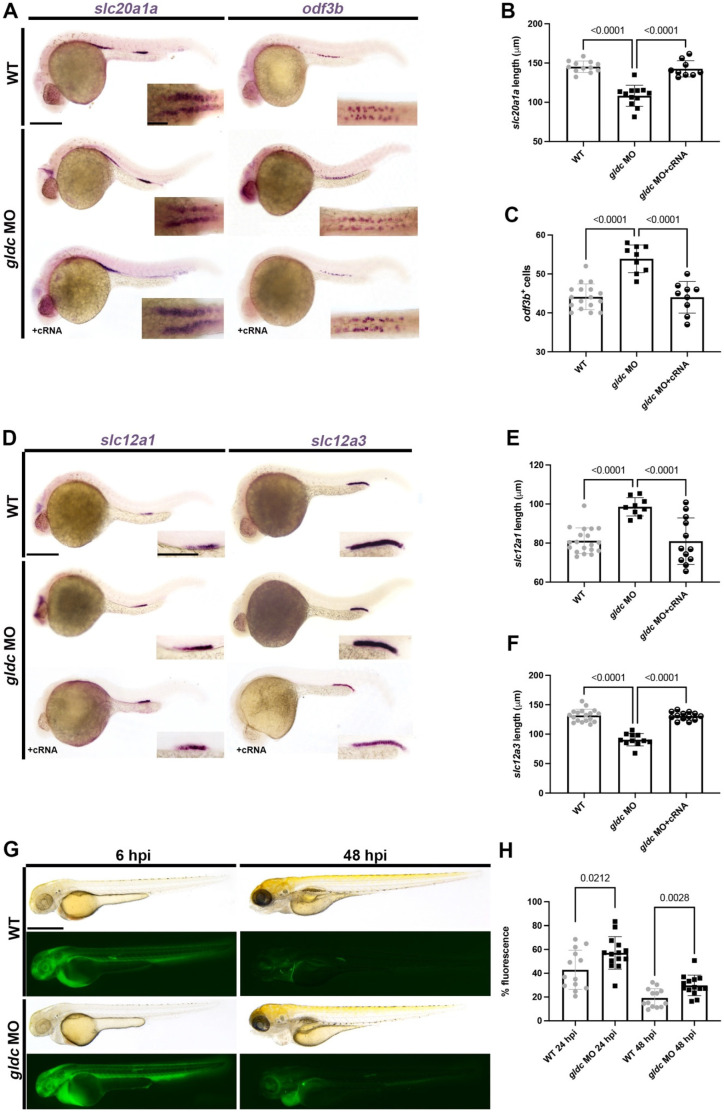
*gldc* is necessary for proper segment patterning. (**A**) WISH of *slc20a1a* and *odf3b* in WT, *gldc* MO, and *gldc* MO + cRNA at 24 hpf. Scale bars = 200 μM (main image) and 50 μM (inset). (**B**) Absolute length quantification of *slc20a1a* domain in control and treatment groups. (**C**) Quantification of *odf3b*^+^ cells in WT, *gldc* MO, and *gldc* MO + cRNA. (**D**) WISH of *slc12a1* and *slc12a3* in WT, *gldc* MO, and *gldc* MO + cRNA at 24 hpf. Scale bars = 200 μM (main image) and 50 μM (inset). (**E**,**F**) Absolute length quantification of *slc12a1* and *slc12a3* domains, respectively. (**G**) Animals were injected with dextran-FITC at 24 hpf, then imaged at 6 hpi and 48 hpi. Scale bar = 400 μM. (**H**) Quantifications of percent fluorescence at 24 hpi and 48 hpi. Percent fluorescence was calculated with 6 hpi fluorescent intensity as baseline. Data are mean ± s.d. quantified for each control and experimental group. Absolute lengths and *odf3b*^+^ cell counts were compared using ANOVA. Percent fluorescence measurements were compared with unpaired *T*-tests.

To assay for different populations within the zebrafish pronephros, we utilized WISH of 24 hpf animals to quantify alterations in *gldc* deficient animals. *gldc* morphants exhibited a reduction in the proximal convoluted tubule (PCT), marked by *slc20a1a* (Figure 3A,B) [19,21]. The *trpm7* domain, which marks transporter cells in the proximal straight tubule (PST) [19,21], did not change upon *gldc* knockdown (Supplemental Figure S2A,B). We also assayed another population in the PST, the multiciliated cells (MCCs) [53,54,55]. MCCs are specialized cells for fluid propulsion [56]. *gldc* dysfunction led to an increased number of cells expressing *odf3b*, an established MCC marker (Figure 3A,C) [35,41,53,55,57]. Next, we analyzed the distal segments, which are homologous to the mammalian thick ascending limb (TAL) and distal convoluted tubule [19,21]. Knockdown of *gldc* caused a significant expansion of the distal early (DE) tubule and a significant reduction of the distal late tubule (DL) (Figure 3D–F). The segment and MCC phenotypes were rescued by co-injection of *gldc* cRNA, validating that *gldc* knockdown is specifically altering segment patterning (Figure 3A–F). There was no significant difference in the total length of the tubule when compared to the body length (Supplemental Figure S2C,D). This indicates that the changes in segments are not due to a decrease in body size, but alterations in the nephron structure.

Due to changes in both live phenotypes and nephron segmentation, we aimed to analyze whether the nephrons were properly functioning in our morphants by assessing renal clearance [42]. At 24 hpf, we performed intramuscular microinjections of dextran-FITC to introduce the fluorescent molecule into circulation (Figure 3G) [58,59,60]. At 6 hpi, we imaged injected animals to establish a baseline fluorescent intensity. The WT and *gldc* morphants were then imaged at 24 hpi and 48 hpi. We found that *gldc* morphants were unable to eliminate the dextran-FITC molecules as efficiently as their WT counterparts. Therefore, the *gldc* morphants had a higher percent fluorescence at both 24 hpi and 48 hpi (Figure 3H). Based on these results, we conclude that *gldc* has a role in proper nephron segmentation and kidney function. Due to the distal expression pattern of *gldc,* we aimed to further analyze the patterning of the distal domain.

### 3.4. Loss of gldc Affects DE and DL Precursor Populations

**Figure 4:**
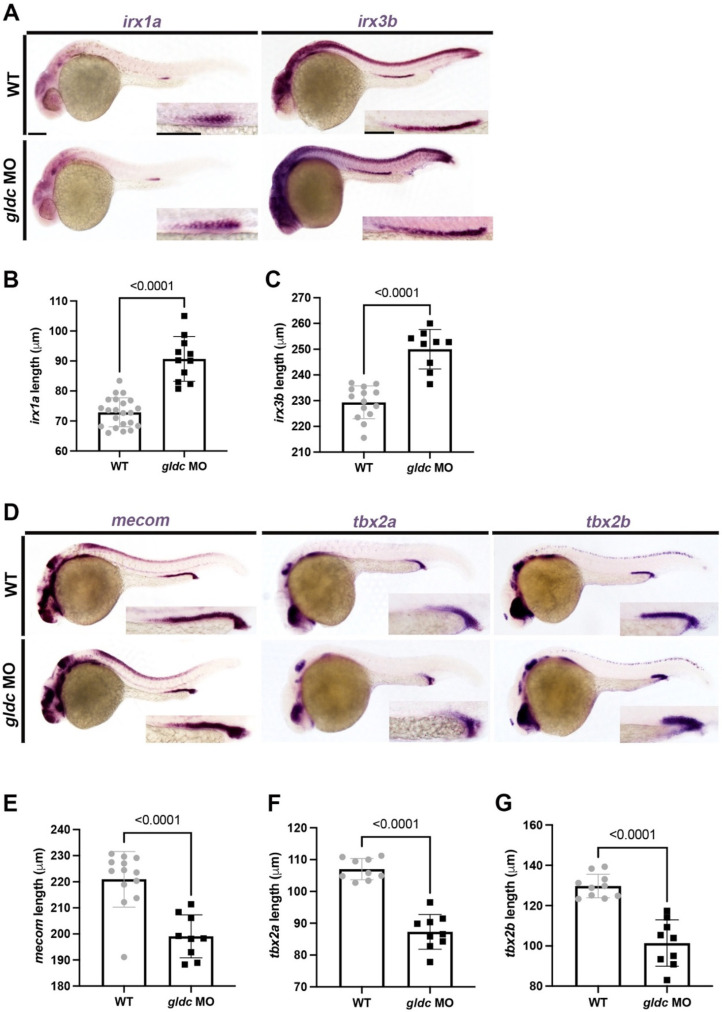
Loss of *gldc* affects DE and DL precursor populations. (**A**) WISH of *irx1a* and *irx3b* in WT and *gldc* morphant embryos at 24 hpf. Scale bars = 100 μM (main image) and 50 μM (inset). (**B**,**C**) Absolute length measurements of *irx1a* and *irx3b* domains. (**D**) WISH of *mecom*, *tbx2a*, and *tbx2b* on WT and *gldc* MO embryos at 24 hpf. Scale bars = 100 μM (main image) and 50 μM (inset). (**E**–**G**) Absolute length measurements of *mecom*, *tbx2a*, and *tbx2b* domains quantified for each control and experimental group. Data are mean ± s.d. Absolute lengths were compared using unpaired *T*-tests.

Previous research has shown that *Iroquois homeobox* transcription factors *irx1a* and *irx3b* are necessary for DE differentiation in the embryonic nephron [21,61,62]. Specifically, *irx3b* works upstream of *irx1a* to eventually pattern *slc12a1*^+^ cells [21,61,62]. To analyze if *gldc* interacts with these factors, we began by utilizing WISH to examine the expression domains of *irx1a* and *irx3b*. We found that upon *gldc* knockdown, there was a significant expansion of both the *irx1a* and *irx3b* domains (Figure 4A–C).

The transcription factor, MECOM, is heavily expressed in the human kidney [63]. In the *Xenopus*, Mecom is an essential part of nephron patterning [64]. More specifically, the loss of function of *mecom* in the zebrafish leads to a decreased DL segment, suggesting an important role in the specification of this domain [49]. Other transcription factors have been reported to be important in distal patterning as well. For example, in *Xenopus*, Tbx2 is responsible for demarcating the boundaries of the pronephros [65]. Loss of function causes an elongation of the nephrons [65]. Conversely, in the zebrafish, *T-box* transcription factors *tbx2a* and *tbx2b* have been found to be sufficient to promote DL differentiation [66]. Additionally, these factors have been noted to be negatively regulated by retinoic acid signaling [66]. Because of the changes in the *slc12a3*^+^ population in our *gldc* morphants, we hypothesized that the *mecom* and *tbx2a/b* domains would be affected as well. To test this, we conducted WISH on 24 hpf animals and measured the domain lengths. We found that *gldc* deficiency caused a significant decrease in the length of the *mecom*, *tbx2a*, and *tbx2b* domains (Figure 4D–G). Therefore, dysfunction of *gldc* perturbs the expression patterns of these factors in renal progenitors, eventually causing alterations in segment domain size.

### 3.5. gldc Morphant Cloacae Exhibit Increased Levels of Apoptosis and Structural Abnormalities

**Figure 5:**
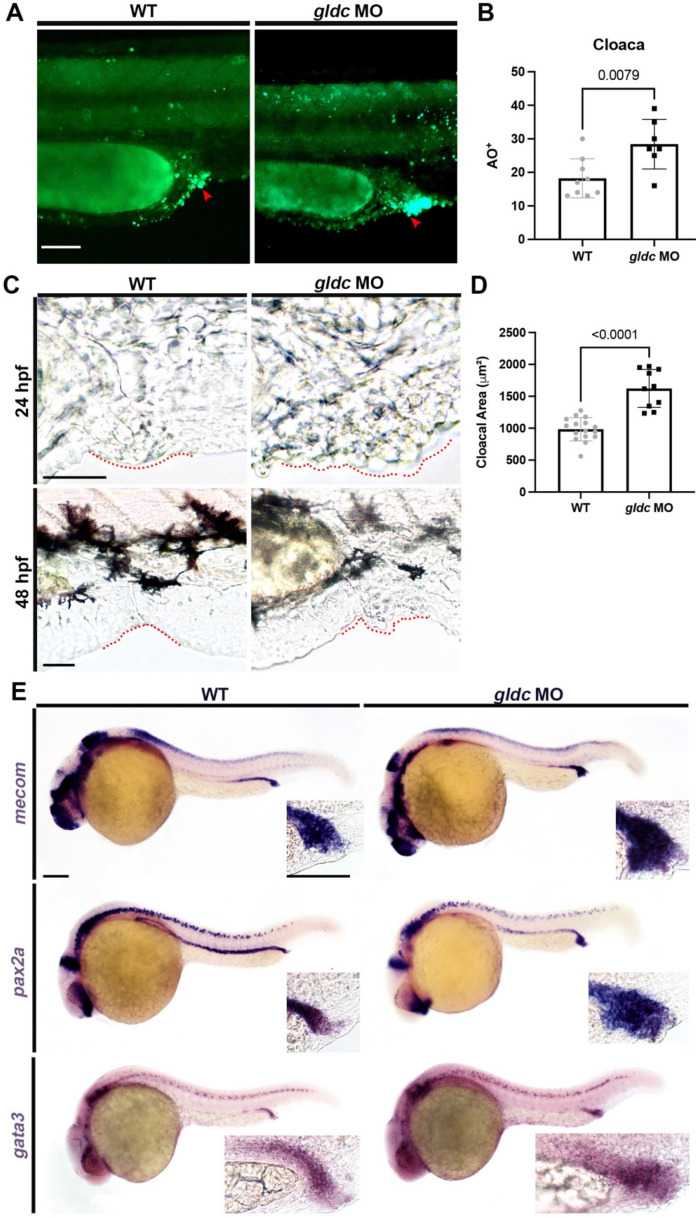
*gldc* morphant cloacae demonstrate increased levels of apoptosis and structural abnormalities. (**A**) Cell death assay of the cloaca in WT and *gldc* morphants at 24 hpf. Scale bar = 50 μM. (**B**) Quantification of AO^+^ cells in the cloaca; red arrowheads indicate example AO^+^ cells. (**C**) Live images of the cloacal region (outlined in red) in WT and *gldc* morphants at 24 hpf and 48 hpf. Scale bars = 50 μM. (**D**) Measurement of the cloacal area in WT and *gldc* MO. (**E**) WISH of *mecom*, *pax2a*, and *gata3* in WT and *gldc* morphants at 24 hpf revealed enlarged cloacal regions. Scale bars = 100 μM. Data are mean ± s.d. AO^+^ cell counts and cloacal area were compared with unpaired *T*-tests.

While conducting the AO assay, we noted the cloaca had a significant increase in apoptosis upon *gldc* knockdown (Figure 5A,B). The cloaca functions as the common opening for the gut and urogenital tract in amphibians, reptiles, birds, and some species of fish [67]. Similar to the kidney, it is derived from ventral mesoderm [67]. Researchers have found that Bmp signaling is essential for the proper formation of the cloaca; however, little is known about the specific genetic signaling cascades that lead to its development [67]. In *gldc* morphants, we found an inability to properly filter out dextran-FITC once in circulation (Figure 3G). Besides alterations in nephron segmentation, another potential explanation for molecular retention is physical abnormalities in the urogenital opening. Upon live imaging, we found the cloacal structure was altered in *gldc* deficient animals (Figure 5C). At 24 hpf and 48 hpf, imaging revealed clusters of cells aggregating at the cloaca (Figure 5C). We utilized WISH of *pax2a*, *mecom*, and *gata3* to better visualize this area (Figure 5E). Next, we measured the area of the cloaca and found that *gldc* deficient animals had an increased area compared to their WT siblings (Figure 5D). Overall, *gldc* deficiency leads to increased apoptosis in the cloaca, alterations in structure, and a significant increase in the cloacal area. The molecular mechanisms underlying morphological changes in *gldc* morphants were unclear. Due to the role of *gldc* in the GCS, we aimed to investigate whether excess glycine caused similar phenotypes to the morphants.

### 3.6. Exogenous Glycine Treatment Recapitulates the Live Morphology and Segment Patterning of gldc Deficient Embryos in a Dose-Dependent Manner

**Figure 6:**
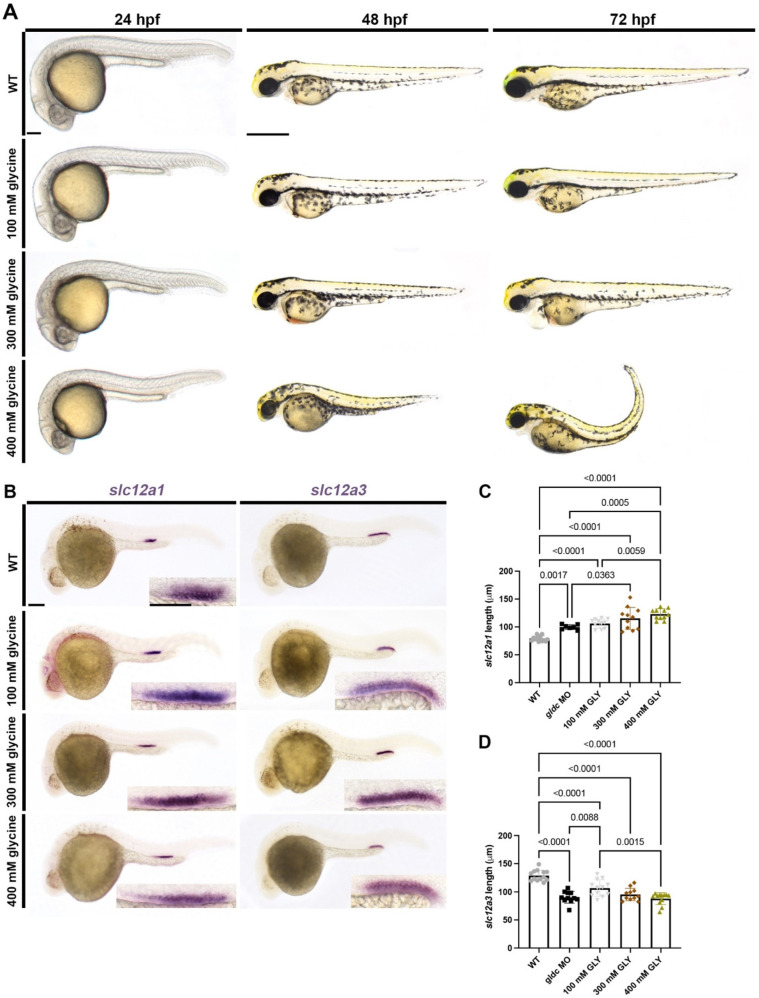
Glycine-treated embryos altered live morphology and segment patterning in a dose-dependent manner, comparable to *gldc* morphants. (**A**) Live images of WT and glycine-treated animals at 24 hpf, 48 hpf, and 72 hpf. Animals were treated with three different concentrations of glycine: 100 mM, 300 mM, and 400 mM. Imaging revealed gray pallor in the brain at 24 hpf in 300 mM and 400 mM animals. Pericardial edema and hydrocephalus became evident in 300 mM zebrafish at 48 hpf and became severe at 72 hpf with aberrant jaw morphology. Mild fluid retention was seen in 100 mM animals at 72 hpf. Zebrafish treated with 400 mM glycine exhibited severe body curvature and overall altered morphology at 72 hpf. Scale bars = 100 μM (24 hpf) and 400 μM (48 hpf and 72 hpf). (**B**) WISH of WT and glycine-treated zebrafish at 24 hpf analyzing *slc12a1* and *slc12a3*. The *slc12a1* domain elongates in glycine-treated animals in a dose-dependent manner. The *slc12a3* domain becomes reduced in the treatment group in a dose-dependent manner as well. Scale bars = 100 μM (main image) and 50 μM (inset). (**C**,**D**) Absolute length quantifications of *slc12a1* and *slc12a3* comparing WT and treatment groups. Data are mean ± s.d. Absolute lengths were compared with ANOVA.

In humans and mice, dysfunctional GLDC/*Gldc* causes increased levels of glycine within the plasma and urine [9,38,68,69]. However, the exact implications excess glycine has on developmental processes are still unknown. Glycine is a simple amino acid that acts as both an excitatory and inhibitory neurotransmitter [70,71]. Additionally, glycine-treated zebrafish have been shown to have altered vasculature development in a dose-dependent manner [36]. To analyze whether glycine has an effect on kidney development, we began by bathing embryos in varying concentrations of glycine beginning at the 50% shield stage. Live imaging at 24 hpf revealed little differences between WT and treated embryos (Figure 6A). At 48 hpf, pericardial edema becomes evident at 300 mM and 400 mM doses (Figure 6A). Multiple phenotypes are observed at 72 hpf, such as pericardial edema, hydrocephalus, and aberrant jaw morphology (Figure 6A). These phenotypes become more severe with higher doses, and at 400 mM, extreme body curvature is present (Figure 6A). We utilized WISH to analyze whether differentiated distal kidney populations were altered in drug-treated animals. We found that glycine-treated embryos exhibit an expanded DE domain and a reduced DL domain in a dose-dependent manner (Figure 6B–D). These trends are consistent with what we noted in our *gldc* deficient embryos, suggesting that *gldc* knockdown causes an increase in glycine which influences tissue development and nephron segment patterning.

## 4. Discussion

Mutations in GLDC account for about 75% of NKH cases, causing a wide variety of symptoms including seizures and developmental delays [4]. Upon investigating *Gldc* deficiency in mice, researchers found animals exhibit dilation of brain ventricles, hydrocephalus, and alterations in brain structures [9,10,12,72]. Additionally, GLDC/*Gldc* has been linked to neural tube defects (NTDs) in humans and mice, further implicating its role in development [9,39,72,73]. Until our study, prenatal lethality in *Gldc* deficient mice prevented the analysis of early developmental processes. Here, our research suggests that we created a more morphologically severe model of *gldc* deficiency in zebrafish.

In a paper by Riché et al. [37], researchers created a *gldc* deficient zebrafish model to assess behavioral defects and metabolic disruptions. While their model had severe motor phenotypes, the overall morphology of the *gldc* mutant animals was relatively normal. Here, we aimed to create a zebrafish model to recapitulate the severe anatomical phenotypes observed in humans and mouse models with Gldc/GLDC genetic alterations. In *Gldc* deficient mice, hydrocephalus and ventriculomegaly were among the most prominent phenotypes [9,12,39,72]. At 48 hpf, initial surveillance of live phenotypes in our morphant zebrafish revealed mild hydrocephalus and pericardial edema, suggesting impaired fluid homeostasis. Additionally, morphants experienced decreased survival rate, similar to human patients and mice [3,9,12]. Surveying at 72 hpf revealed malformations of jaw development in *gldc* deficient embryos. In a study of NKH patients, ~30% of the test subjects presented with micrognathia [46]. Utilizing Alcian Blue, we found disturbances in the craniofacial cartilage. Specifically, there were disruptions in the Meckel’s cartilage and a decreased number of pharyngeal arches. We aimed to further analyze the patterning of the jaw and validate our model through analysis of neurological phenotypes.

Zebrafish craniofacial cartilage is patterned by cranial neural crest cells that migrate from the rhombomere regions anteriorly [30]. The migrating neural crest cells expressing *dlx2* in *gldc* morphants were oriented in a disorganized pattern compared to their WT counterparts. Disorganization in migration may explain the abnormalities in craniofacial cartilage. We also aimed to analyze the rhombomeres as they are a vital aspect of brain development and house cranial neural crest cells [30]. In vertebrates, rhombomeres are a segmented phase of the neural tube that later gives rise to the hindbrain [74]. Upon WISH, we found rhombomere patterning in our morphants was altered. Analysis of *mecom*, found to be expressed in rhombomere four, revealed an atypical expression pattern [64]. Other rhombomeres were affected, as the location of rhombomere 3 (marked by *krox20*), shifted posteriorly.

NKH patients exhibit diffusion restriction in the cerebellum due to fluid accumulation and decreased myelination in the pons over time [3,8,11,75]. These areas are developed from the rhombomeres [76], which were altered in our *gldc* morphants. Additional phenotypes noted in MRIs are cortex atrophy, widened ventricles, and a thinning of the corpus callosum [3,8,11,75]. Murine models of *Gldc* deficiency exhibit hydrocephalus, ventriculomegaly, neural tube defects, impaired fluid flow, and aqueduct stenosis [9,12,72,76]. In *gldc* morphants, ventriculomegaly was evident, recapitulating what has been noted in *Gldc* deficient mice and NKH patients. We were curious how *gldc* knockdown was causing brain anatomy alterations in our morphants. Upon further research, we found that GLDC has many important roles including regulating glycine levels and glutathione production in the liver [77]. Glycine is a simple amino acid that enhances anti-oxidative functions, improves protein synthesis, and is a building block for a variety of compounds, including glutathione [78]. Glutathione is imperative to controlling immune responses, breaking down nutrients, and providing protection against reactive oxygen species (ROS) [77]. Because GLDC affects a wide variety of cellular processes, we decided to start by investigating cell death in knockdown animals. We visualized apoptosis patterns in our morphants with an Acridine Orange (AO) assay. In *gldc* morphants, we found an increase in apoptosis in the brain, and interestingly, we noted the same trend in the pronephric region. This apoptosis trend in the pronephros coupled with impaired fluid dynamics suggested abnormal kidney development.

The kidney is a vital organ that functions to transport and uptake solutes, eliminate waste, and maintain fluid homeostasis. In humans and mice, GLDC/*Gldc* is heavily expressed in the embryonic kidney [43,44,45]. Until now, *gldc* expression in the zebrafish has only been depicted in the central nervous system (CNS) [37,79]. However, across several time points, we found *gldc* transcripts expressed within the distal portion of the pronephros. Due to the conserved expression within vertebrates and consistent expression over time in the kidney, we hypothesized that *gldc* has an important role in nephron patterning. Upon WISH analysis, we found that *gldc* deficient animals had a decreased PCT domain length, which is the main region where glycine uptake occurs [80]. Another proximal cell type, MCCs, increased in number as a result of *gldc* knockdown. MCCs are important in maintaining proper fluid homeostasis as they help propel fluid along the tubule [35,41,53,54,55]. The distal segments, vital for ion uptake and water reabsorption, were affected by *gldc* knockdown. The DE tubule increased in length, while the DL tubule decreased in length, creating an imbalance in the specified cells in this region. Segment changes can lead to impaired kidney performance. To assess whether these alterations in differentiated cell populations were affecting kidney function, we utilized dextran conjugated FITC to visualize fluid retention. We found that *gldc* morphants were unable to excrete the dextran FITC as efficiently as WT animals. These results are similar to humans with GLDC deficiency, as kidney defects have been briefly described in addition to a wide variety of other symptoms [47,81,82]. To dissect how *gldc* knockdown perturbs the distal genetic pathway, we analyzed factors that pattern these tubules.

The DE and DL domains in the zebrafish are analogous to the mammalian thick ascending limb (TAL) and distal convoluted tubule (DCT), respectively [19,20]. The TAL and DCT have various functions but are vital to water and electrolyte balance and acid-base homeostasis [83,84]. The GCS functions on the mitochondrial membrane, and DCT cells are rich in mitochondria, making them relevant to our studies [84]. The genetic cascades that lead to the differentiation of the DE and DL are not well understood. However, there are transcription factors necessary for the proper formation of these domains. In the DE, these include *Iroquois homeobox* transcription factors, *irx1a* and *irx3b* [21,62,85]. WISH analysis on *gldc* morphants revealed an increase in length in the domains expressing *irx1a* and *irx3b*. Patterning of the DL domain includes interactions between *mecom* and *tbx2a/b* transcription factors [49,66,86]. We utilized WISH to assess the spatiotemporal domains of *mecom*, *tbx2a*, and *tbx2b* in WT and *gldc* morphants at 24 hpf. In *gldc* deficient animals, the number of cells expressing these transcription factors was diminished, correlating with the decrease in the DL domain. These upstream effects suggest that *gldc* has a role in earlier patterning processes of the nephron.

As each segment of the nephron carries out a specific job, changes in domain lengths can impair kidney function. However, morphological changes can also affect the excretion of waste. When assaying apoptosis trends in *gldc* morphants, we noted a large increase in death in the collecting duct/cloaca region. The cloaca is the common opening for the intestinal, urinary, and genital systems [67]. Live imaging revealed alteration in the cloacal structure as there was an aggregation of cells around the opening of the duct. Quantification of these phenotypes through area measurements revealed a significant increase in the area of the cloaca upon knockdown of *gldc*. Not much is known about cloaca development in zebrafish, however, sustained Bmp signaling is needed for proper formation [67]. Recently, *slc20a1a* has been identified as a candidate gene for cloacal exstrophy as knockdown animals exhibit cloacal anomalies with abnormal urinary outflow [87]. In *gldc* deficient animals, this morphological abnormality may block fluid excretion which provides another possible explanation for the abnormal fluid homeostasis. Further analysis would be needed to determine the cause of altered cell dynamics and patterning in this region, as *gldc* has a variety of important roles, including glycine regulation.

GLDC is a vital part of the GCS, and when dysfunctional, causes an increase in glycine globally [88]. Glycine is a neurotransmitter that has been shown to be vital to neuronal migration, MAPK signaling, and mitochondrial function in the brain [89,90,91]. In zebrafish, glycine-treated animals exhibited developmental defects in the heart, liver, and brain [92]. Another study determined that glycine treatment had dose-dependent biphasic effects on vasculature development in zebrafish [36]. These vascular alterations were impacted by levels of PI3K/Akt/mTor signaling [93], and several case reports have reported vascular abnormalities, namely hypertension, in some NKH patients [94,95,96,97]. Interestingly, there is a well-established relationship between the balance of renal and vascular progenitor fate choice and cell survival in developing zebrafish [98,99,100]. Here, our studies reveal nephron alterations in the process of segmentation consequent to elevated glycine levels. In NKH patients, a relationship has been reported between the increase in glycine concentration and the increase in the severity of symptoms [2]. We found a similar trend, as glycine-treated embryos increased in morphological severity with higher doses. Survivability also decreased as the concentration of glycine increased. In the kidney, glycine has been shown to increase renal plasma flow and glomerular filtration rate [80,101]. Glycine also affects ion uptake as it inhibits proximal and distal tubule Na+ reabsorption in rats [102]. In our treated animals, we found nephron segmentation was altered in the same fashion as our *gldc* morphants. We concluded that *gldc* knockdown causes an increase in glycine within the animal which impacts the genetic patterning pathways of the kidney. Further studies are needed to assess the physiological consequences of these developmental phenotypes due to exposure to high glycine during organogenesis.

## 5. Conclusions

**Figure 7:**
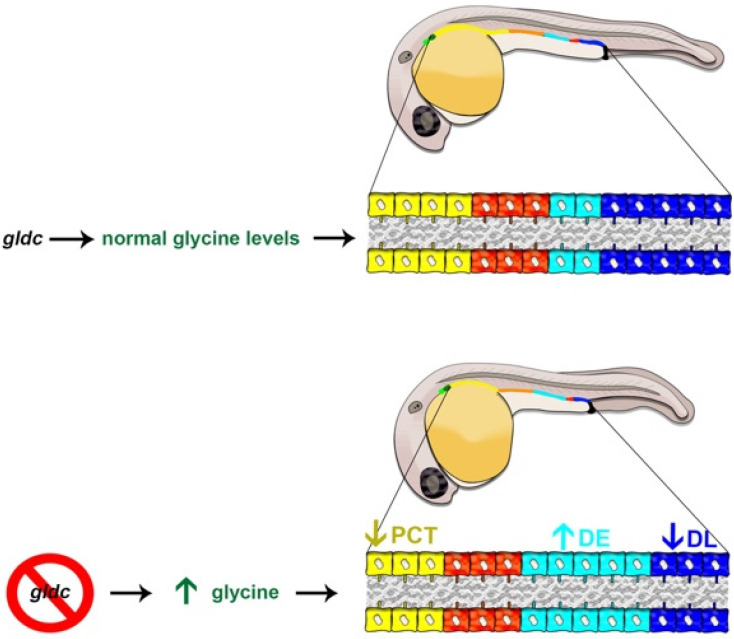
Nephrogenesis in the WT zebrafish embryo compared to *gldc* deficiency which leads to lethal elevated glycine levels. (**Top**) At 24 hpf, when the nephron segment pattern is established, WT embryos exhibit nephrons tubules with a series of four segments: the PCT (yellow-colored cells), PST (orange-colored cells), DE (turquoise-colored cells) and DL (dark blue colored cells). (**Bottom**) nephron tubule segment populations are altered in *gldc* deficient zebrafish, such that the PCT segment is decreased in size (arrow down), while the DE segment population is expanded (arrow up) and the DL segment is decreased (arrow down). These alterations correlate with changes in the expression of essential renal progenitor genes, observations that provide a crucial foundation for future studies to elucidate the underlying molecular mechanisms of these nephrogenesis defects.

Here, we have characterized a novel zebrafish model of *gldc* deficiency that displays classic NKH hallmarks of neural and craniofacial malformations, and discovered new features including several renal and urogenital tract malformations. We show that *gldc* is essential for determining patterning events of renal progenitors that give rise to the nephron functional units (Figure 7), which will be useful for understanding the genetic basis of kidney diseases [103]. These insights, along with this original *gldc* deficiency model, provide exciting new opportunities to expand our understanding of the roles of glycine metabolism in ontogeny.

## Data Availability

All data are contained in this article and the Supplementary Materials.

## Supplementary Materials

The following supporting information can be downloaded at: https://www.mdpi.com/article/10.3390/biomedicines10123220/s1, Figure S1: Design and validation of *gldc* knockdown tools; Figure S2: Additional nephron analysis in *gldc* deficient embryos.
