# Supplementary material for: gldc Is Essential for Renal Progenitor Patterning during Kidney Development
Source: Biomedicines. 2022 Dec 12;10(12):3220. doi: 10.3390/biomedicines10123220 (PMC9776136; doi:10.3390/biomedicines10123220)
Supplement: Supplementary file 1 [file biomedicines-10-03220-s001.zip › biomedicines-2046018-supplementary.pdf]

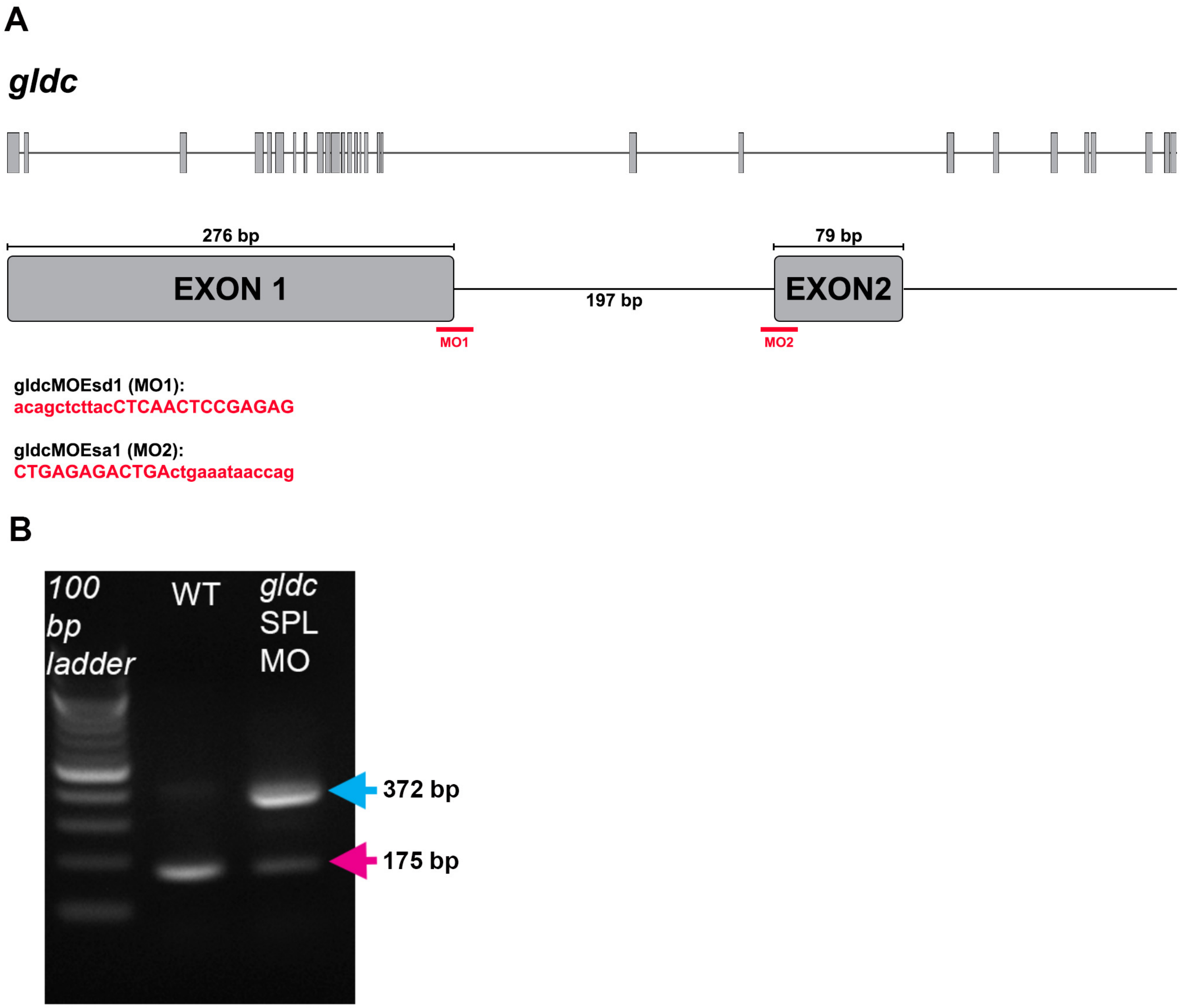

Supplemental Figure S1. Design and validation of *gldc* knockdown tools. (A) Gene map of *gldc* in zebrafish exhibit the location of the splice blocking morpholinos utilized with red underlines. **(B)** WT and *gldc* MO DNA was run on an agarose gel. The WT DNA ran to 175 base pairs as expected, and the *gldc* MO DNA exhibited two separate bands at 372 base pairs and 175 base pairs. The bands were cut and gel purified before sent to sequencing. Sequencing analysis confirmed *gldc* MOs retained intron 1 which encoded a premature stop codon.

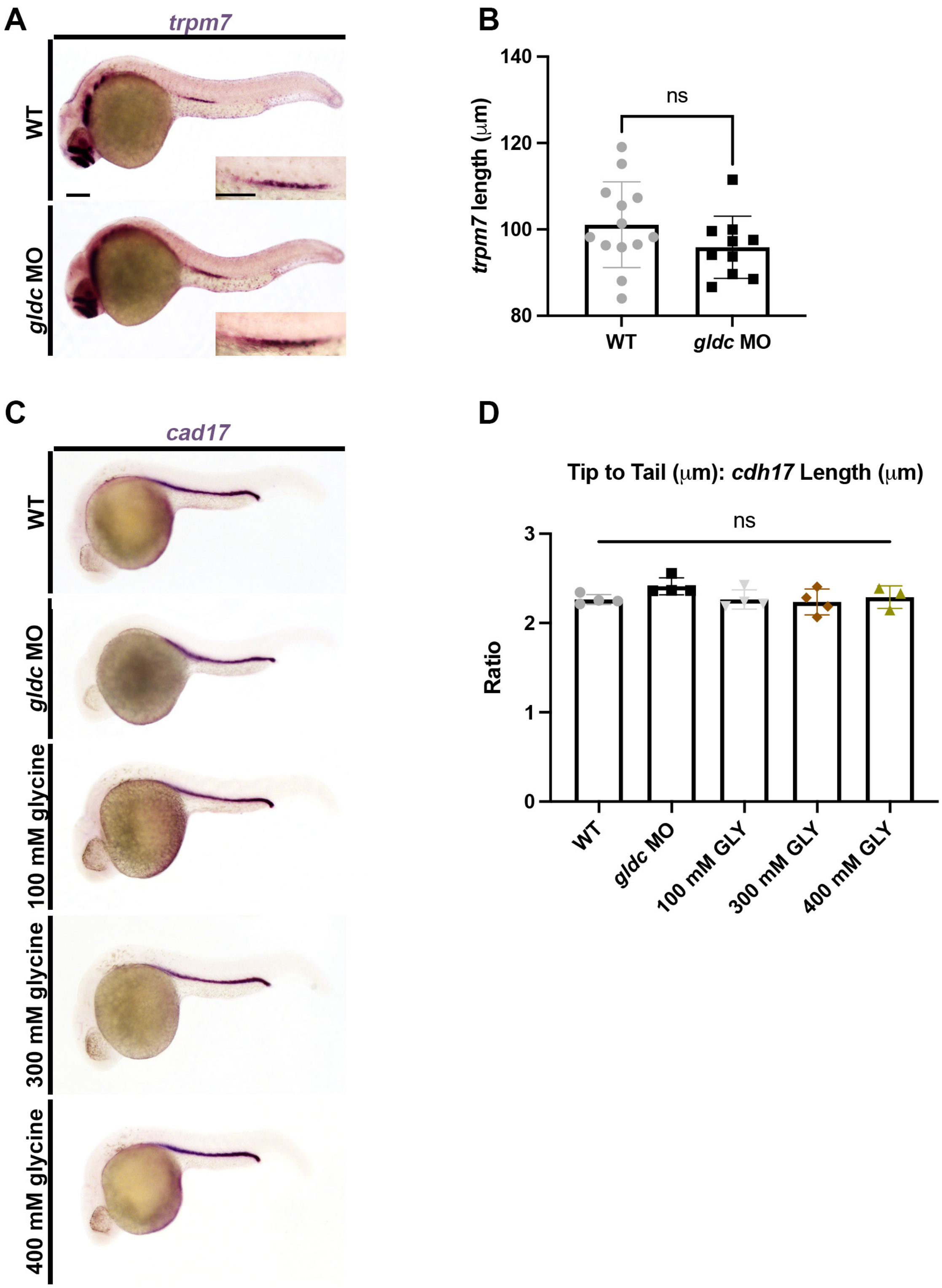

Supplemental Figure S2. Additional nephron analysis in *gldc* deficient embryos. (A) WISH of *trpm7* in WT and *gldc* MO at 24 hpf. Scale bars= 200 μM (main image) and 50 μM (inset). **(B)** Absolute length quantifications of *trpm7* domain in control and *gldc* MO groups indicating no significant difference in length. **(C)** WISH of *cdh17* in WT, *gldc* MO, and treated animals at 24 hpf. Scale bar= 200 μM. **(D)** Ratios were calculated to compare tip to tail length versus the length of the entire tubule. There was no significant difference in the ratios between WT and treatment groups. n≥8 for the control and treatment groups. Data are mean±s.d. Absolute lengths and ratios were compared with ANOVA.
